# Supplementary material for: Association between oral microbiome and seven types of cancers in East Asian population: a two-sample Mendelian randomization analysis
Source: Front Mol Biosci. 2023 Nov 21;10:1327893. doi: 10.3389/fmolb.2023.1327893 (PMC10702768; doi:10.3389/fmolb.2023.1327893)
Supplement: Supplementary file 1 [file DataSheet1.ZIP › Supplementary Materials/MR plots of saliva/saliva═╝/colorectal cancer/pheno.2831_to_colorectal cancer_leave_one_out.pdf]

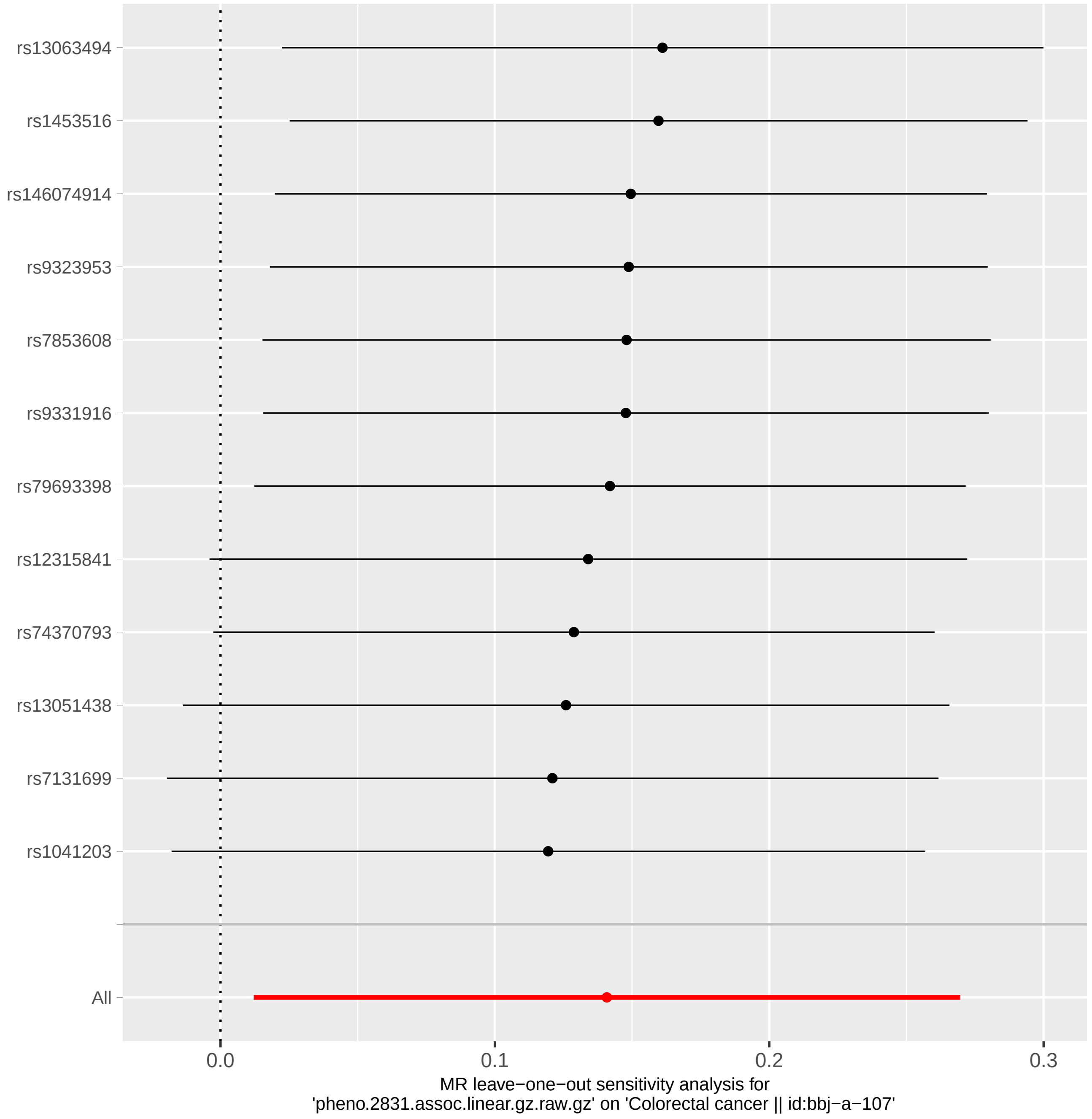

rs13063494
rs1453516
rs146074914
rs9323953
rs7853608
rs9331916
rs79693398
rs12315841
rs74370793
rs13051438
rs7131699
rs1041203
All
0.0
0.1
0.2
0.3
MR leave−one−out sensitivity analysis for
'pheno.2831.assoc.linear.gz.raw.gz' on 'Colorectal cancer || id:bbj−a−107'
